# 6′-Methyl-1′,2′,3′,4′-tetra­hydro­spiro­cyclo­hexane-2′-quinazolin-4′-one

**DOI:** 10.1107/S1600536809014111

**Published:** 2009-04-22

**Authors:** Zhang Ling, Daxin Shi, Fan Yanqiu, Xia Wei, Jiarong Li

**Affiliations:** a School of Chemical Engineering and Environment, Beijing Institute of Technology, Beijing 100081, People’s Republic of China

## Abstract

The title compound, C_14_H_18_N_2_O, was synthesized by the reaction of cyclo­hexa­none and 2-amino-5-methyl­benzonitrile. In the mol­ecule, the cyclo­hexane ring displays a chair conformation, whereas the 1,3-diaza­cyclo­hexane moiety of the bicyclic system has a sofa conformation with the spiro C atom displaced by 0.603 (2) Å from the rest of the atoms of the 1,3-diaza­cyclo­hexane ring [planar within 0.052 (2) Å]. Mol­ecules are linked into centrosymmetric dimers *via* N—H⋯O hydrogen bonds.

## Related literature

For medicinal and biological properties of dihydro­quinazolin-4(3*H*)-one derivatives, see: Jackson *et al.* (2007 ▶); Shi *et al.* (2003 ▶, 2004 ▶).
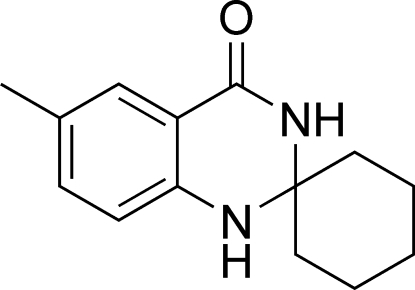


## Experimental

### 

#### Crystal data

C_14_H_18_N_2_O
*M* *_r_* = 230.30
Monoclinic, 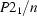

*a* = 9.4077 (19) Å
*b* = 11.853 (2) Å
*c* = 11.067 (2) Å
β = 106.44 (3)°
*V* = 1183.6 (4) Å^3^
*Z* = 4
Mo *K*α radiation
μ = 0.08 mm^−1^
*T* = 113 K
0.28 × 0.24 × 0.20 mm

#### Data collection

Rigaku Saturn CCD area-detector diffractometer
Absorption correction: multi-scan (*CrystalClear*; Rigaku/MSC, 2005 ▶) *T*_min_ = 0.977, *T*_max_ = 0.984
14356 measured reflections
2810 independent reflections
2346 reflections with *I* > 2σ(*I*)
*R*_int_ = 0.034

#### Refinement

*R*[*F* ^2^ > 2σ(*F* ^2^)] = 0.039
*wR*(*F* ^2^) = 0.109
*S* = 1.09
2810 reflections
163 parameters
H atoms treated by a mixture of independent and constrained refinement
Δρ_max_ = 0.30 e Å^−3^
Δρ_min_ = −0.26 e Å^−3^

### 

Data collection: *CrystalClear* (Rigaku/MSC, 2005 ▶); cell refinement: *CrystalClear*; data reduction: *CrystalClear*; program(s) used to solve structure: *SHELXS97* (Sheldrick, 2008 ▶); program(s) used to refine structure: *SHELXL97* (Sheldrick, 2008 ▶); molecular graphics: *SHELXTL* (Sheldrick, 2008 ▶); software used to prepare material for publication: *SHELXTL*.

## Supplementary Material

Crystal structure: contains datablocks global, I. DOI: 10.1107/S1600536809014111/ya2085sup1.cif

Structure factors: contains datablocks I. DOI: 10.1107/S1600536809014111/ya2085Isup2.hkl

Additional supplementary materials:  crystallographic information; 3D view; checkCIF report

## Figures and Tables

**Table 1:** Hydrogen-bond geometry (Å, °)

*D*—H⋯*A*	*D*—H	H⋯*A*	*D*⋯*A*	*D*—H⋯*A*
N2—H2*A*⋯O1^i^	0.901 (16)	2.058 (16)	2.9563 (13)	174.5 (13)

Symmetry code: (i) 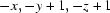.

**Fig. 1.**
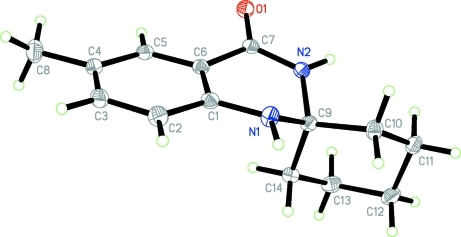
Molecular structure of the title compound with thermal displacement ellipsoids drawn at the 50% probability level; the H atoms are represented as small circles of arbitrary radius.

**Fig. 2.**
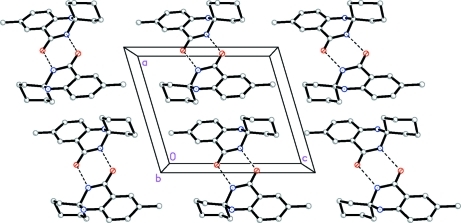
The crystal packing of the title compound, viewed down the b axis; H-bonds are shown as dashed lines.

## supplementary crystallographic information

### Comment

Derivatives of dihydroquinazolin-4(3*H*)-one are valuable synthetic intermediates featuring common structural motif found in a variety of compounds with interesting medicinal and biological properties (Shi *et al.*, 2004; Jackson *et al.*, 2007).

In the molecule of the title compound (Fig. 1) the cyclohexane ring displays a regular chair conformation, whereas, the 1,3-diazacyclohexane moiety of the bicyclic system has a sofa conformation with the C9 atom displaced by 0.603 (2) Å from the rest of the atoms of the 1,3-diazacyclohexane ring (planar within 0.052 (2) Å).

Molecules in crystal are linked into centrosymmetric dimers *via* N2—H2A···O1^i^ [symmetry code (i): -*x*, 1 - *y*, 1 - *z*] bond (Fig. 2).

The molecular geometry and overall crystal structure of the title compound are quite similar to those observed in the structure of its close analog which lacks the methyl substituent in position 6 of the tetrahydroquinazolinone system (Shi *et al.*, 2003).

### Experimental

A solution of 2-amino-5-methylbenzonitrile (10 mmol) and zinc chloride (10 mmol) in cyclohexanone (2 ml) was refluxed for 2 h. The reaction mixture was cooled to room temperature and poured into 20 ml of water (previously cooled to 20°); it was then filtered *in vacuo* to give the title compound. The product was recrystallizated from ethanol to give colorless crystalline powder. m.p. 527–528 K; IR (KBr): 3367 (N—H), 3028, 2936 (C—H), 1648 (C=O) cm^-1^; ^1H^-NMR(DMSO, p.p.m.): 1.25–1.78 (10*H*, m), 2.35(3*H*, s) 6.63 (1*H*, m), 6.87 (1*H*, d), 6.91 (1*H*, s), 7.55 (1*H*, d), 8.06(1*H*, s). 50 mg of the obtained product was dissolved in ethyl acetate (5 ml) and the solution was kept at room temperature for 4 days to give colorless single crystals.

### Refinement

The H atoms bonded to C were included in the riding model approximation with C—H distances 0.95–0.99 Å, and with *U*_iso_=1.2*U*_eq_ or 1.5*U*_eq_ (for methyl H atoms). The H atoms bonded to N were located in the difference Fourier map and refined isotropically [N1—H1 0.89 (2); N2—H2A 0.90 (2)].

### Crystal data

**Table:**

C_14_H_18_N_2_O	*F*(000) = 496
*M**_r_* = 230.30	*D*_x_ = 1.292 Mg m^−^^3^
Monoclinic, *P*2_1_/*n*	Mo *K*α radiation, λ = 0.71073 Å
Hall symbol: -P 2yn	Cell parameters from 3810 reflections
*a* = 9.4077 (19) Å	θ = 1.7–27.9°
*b* = 11.853 (2) Å	µ = 0.08 mm^−^^1^
*c* = 11.067 (2) Å	*T* = 113 K
β = 106.44 (3)°	Cube, colorless
*V* = 1183.6 (4) Å^3^	0.28 × 0.24 × 0.20 mm
*Z* = 4	

### Data collection

**Table:**

Rigaku Saturn CCD area-detector diffractometer	2810 independent reflections
Radiation source: rotating anode	2346 reflections with *I* > 2σ(*I*)
confocal	*R*_int_ = 0.034
Detector resolution: 7.31 pixels mm^-1^	θ_max_ = 27.9°, θ_min_ = 2.5°
ω and φ scans	*h* = −12→12
Absorption correction: multi-scan (*CrystalClear*; Rigaku/MSC, 2005)	*k* = −15→15
*T*_min_ = 0.977, *T*_max_ = 0.984	*l* = −14→14
14356 measured reflections	

### Refinement

**Table:**

Refinement on *F*^2^	Primary atom site location: structure-invariant direct methods
Least-squares matrix: full	Secondary atom site location: difference Fourier map
*R*[*F*^2^ > 2σ(*F*^2^)] = 0.039	Hydrogen site location: inferred from neighbouring sites
*wR*(*F*^2^) = 0.109	H atoms treated by a mixture of independent and constrained refinement
*S* = 1.09	*w* = 1/[σ^2^(*F*_o_^2^) + (0.0601*P*)^2^ + 0.2329*P*] where *P* = (*F*_o_^2^ + 2*F*_c_^2^)/3
2810 reflections	(Δ/σ)_max_ = 0.003
163 parameters	Δρ_max_ = 0.30 e Å^−^^3^
0 restraints	Δρ_min_ = −0.26 e Å^−^^3^

### Special details

**Table:**

Geometry. All e.s.d.'s (except the e.s.d. in the dihedral angle between two l.s. planes) are estimated using the full covariance matrix. The cell e.s.d.'s are taken into account individually in the estimation of e.s.d.'s in distances, angles and torsion angles; correlations between e.s.d.'s in cell parameters are only used when they are defined by crystal symmetry. An approximate (isotropic) treatment of cell e.s.d.'s is used for estimating e.s.d.'s involving l.s. planes.
Refinement. Refinement of *F*^2^ against ALL reflections. The weighted *R*-factor *wR* and goodness of fit *S* are based on *F*^2^, conventional *R*-factors *R* are based on *F*, with *F* set to zero for negative *F*^2^. The threshold expression of *F*^2^ > σ(*F*^2^) is used only for calculating *R*-factors(gt) *etc*. and is not relevant to the choice of reflections for refinement. *R*-factors based on *F*^2^ are statistically about twice as large as those based on *F*, and *R*- factors based on ALL data will be even larger.

### Fractional atomic coordinates and isotropic or equivalent isotropic displacement parameters (Å^2^)

**Table:**

	*x*	*y*	*z*	*U*_iso_*/*U*_eq_	
O1	0.01942 (8)	0.42294 (7)	0.36579 (7)	0.0182 (2)	
N1	0.32706 (11)	0.24403 (8)	0.61566 (9)	0.0167 (2)	
N2	0.16255 (10)	0.39739 (8)	0.56703 (8)	0.0144 (2)	
C1	0.31997 (12)	0.22860 (9)	0.49040 (10)	0.0153 (2)	
C2	0.39770 (12)	0.14331 (9)	0.44884 (11)	0.0191 (2)	
H2	0.4588	0.0928	0.5078	0.023*	
C3	0.38557 (12)	0.13253 (9)	0.32154 (11)	0.0197 (2)	
H3	0.4379	0.0736	0.2946	0.024*	
C4	0.29853 (12)	0.20585 (9)	0.23162 (10)	0.0180 (2)	
C5	0.21867 (12)	0.28808 (9)	0.27333 (10)	0.0163 (2)	
H5	0.1573	0.3381	0.2139	0.020*	
C6	0.22636 (11)	0.29905 (9)	0.40055 (10)	0.0143 (2)	
C7	0.12913 (11)	0.37917 (9)	0.44220 (10)	0.0141 (2)	
C8	0.29082 (14)	0.19553 (11)	0.09415 (11)	0.0268 (3)	
H8A	0.2017	0.2339	0.0431	0.040*	
H8B	0.2869	0.1156	0.0707	0.040*	
H8C	0.3789	0.2304	0.0793	0.040*	
C9	0.30268 (11)	0.35816 (9)	0.65516 (9)	0.0142 (2)	
C10	0.28781 (13)	0.35125 (9)	0.78910 (10)	0.0176 (2)	
H10A	0.1961	0.3095	0.7872	0.021*	
H10B	0.3726	0.3080	0.8423	0.021*	
C11	0.28289 (12)	0.46671 (10)	0.84905 (10)	0.0192 (2)	
H11A	0.2840	0.4566	0.9381	0.023*	
H11B	0.1895	0.5055	0.8046	0.023*	
C12	0.41467 (13)	0.53975 (10)	0.84304 (10)	0.0215 (3)	
H12A	0.4071	0.6148	0.8799	0.026*	
H12B	0.5081	0.5040	0.8927	0.026*	
C13	0.41659 (13)	0.55324 (10)	0.70664 (10)	0.0204 (3)	
H13A	0.5013	0.6013	0.7029	0.024*	
H13B	0.3242	0.5906	0.6574	0.024*	
C14	0.43013 (12)	0.43771 (9)	0.64986 (10)	0.0169 (2)	
H14A	0.5257	0.4030	0.6963	0.020*	
H14B	0.4303	0.4474	0.5610	0.020*	
H2A	0.1067 (16)	0.4493 (13)	0.5919 (13)	0.027 (4)*	
H1	0.3942 (18)	0.2055 (13)	0.6744 (14)	0.031 (4)*	

### Atomic displacement parameters (Å^2^)

**Table:**

	*U*^11^	*U*^22^	*U*^33^	*U*^12^	*U*^13^	*U*^23^
O1	0.0160 (4)	0.0208 (4)	0.0164 (4)	0.0053 (3)	0.0024 (3)	0.0005 (3)
N1	0.0198 (5)	0.0136 (5)	0.0159 (5)	0.0048 (4)	0.0040 (4)	0.0011 (3)
N2	0.0131 (4)	0.0143 (4)	0.0157 (4)	0.0030 (3)	0.0039 (3)	−0.0006 (3)
C1	0.0143 (5)	0.0135 (5)	0.0181 (5)	−0.0014 (4)	0.0048 (4)	−0.0013 (4)
C2	0.0187 (5)	0.0154 (5)	0.0226 (6)	0.0040 (4)	0.0045 (4)	−0.0011 (4)
C3	0.0175 (5)	0.0178 (5)	0.0247 (6)	0.0007 (4)	0.0073 (4)	−0.0065 (4)
C4	0.0153 (5)	0.0199 (6)	0.0192 (5)	−0.0034 (4)	0.0058 (4)	−0.0052 (4)
C5	0.0135 (5)	0.0171 (5)	0.0176 (5)	−0.0014 (4)	0.0034 (4)	−0.0018 (4)
C6	0.0124 (5)	0.0126 (5)	0.0178 (5)	−0.0014 (4)	0.0042 (4)	−0.0015 (4)
C7	0.0124 (5)	0.0128 (5)	0.0172 (5)	−0.0012 (4)	0.0045 (4)	0.0005 (4)
C8	0.0291 (6)	0.0327 (7)	0.0198 (6)	0.0046 (5)	0.0090 (5)	−0.0052 (5)
C9	0.0140 (5)	0.0135 (5)	0.0142 (5)	0.0020 (4)	0.0027 (4)	−0.0001 (4)
C10	0.0207 (5)	0.0178 (5)	0.0148 (5)	0.0019 (4)	0.0056 (4)	0.0020 (4)
C11	0.0196 (5)	0.0226 (6)	0.0144 (5)	0.0036 (4)	0.0031 (4)	−0.0017 (4)
C12	0.0196 (6)	0.0231 (6)	0.0186 (5)	0.0002 (5)	0.0004 (4)	−0.0059 (4)
C13	0.0196 (5)	0.0175 (6)	0.0224 (6)	−0.0032 (4)	0.0033 (4)	−0.0012 (4)
C14	0.0140 (5)	0.0183 (5)	0.0180 (5)	−0.0009 (4)	0.0042 (4)	−0.0003 (4)

### Geometric parameters (Å, °)

**Table:**

O1—C7	1.2472 (13)	C8—H8B	0.9800
N1—C1	1.3810 (14)	C8—H8C	0.9800
N1—C9	1.4596 (14)	C9—C10	1.5299 (14)
N1—H1	0.893 (16)	C9—C14	1.5394 (15)
N2—C7	1.3446 (13)	C10—C11	1.5273 (16)
N2—C9	1.4757 (14)	C10—H10A	0.9900
N2—H2A	0.901 (16)	C10—H10B	0.9900
C1—C2	1.3995 (15)	C11—C12	1.5289 (16)
C1—C6	1.4022 (15)	C11—H11A	0.9900
C2—C3	1.3867 (15)	C11—H11B	0.9900
C2—H2	0.9500	C12—C13	1.5232 (16)
C3—C4	1.3982 (17)	C12—H12A	0.9900
C3—H3	0.9500	C12—H12B	0.9900
C4—C5	1.3870 (15)	C13—C14	1.5269 (15)
C4—C8	1.5074 (15)	C13—H13A	0.9900
C5—C6	1.3953 (14)	C13—H13B	0.9900
C5—H5	0.9500	C14—H14A	0.9900
C6—C7	1.4797 (14)	C14—H14B	0.9900
C8—H8A	0.9800		
			
C1—N1—C9	117.15 (9)	N2—C9—C10	110.35 (9)
C1—N1—H1	119.1 (10)	N1—C9—C14	111.48 (9)
C9—N1—H1	113.2 (10)	N2—C9—C14	109.97 (8)
C7—N2—C9	122.26 (9)	C10—C9—C14	110.82 (9)
C7—N2—H2A	115.9 (9)	C11—C10—C9	113.28 (9)
C9—N2—H2A	120.1 (9)	C11—C10—H10A	108.9
N1—C1—C2	122.97 (10)	C9—C10—H10A	108.9
N1—C1—C6	118.37 (10)	C11—C10—H10B	108.9
C2—C1—C6	118.61 (10)	C9—C10—H10B	108.9
C3—C2—C1	120.00 (10)	H10A—C10—H10B	107.7
C3—C2—H2	120.0	C10—C11—C12	111.34 (9)
C1—C2—H2	120.0	C10—C11—H11A	109.4
C2—C3—C4	121.94 (10)	C12—C11—H11A	109.4
C2—C3—H3	119.0	C10—C11—H11B	109.4
C4—C3—H3	119.0	C12—C11—H11B	109.4
C5—C4—C3	117.59 (10)	H11A—C11—H11B	108.0
C5—C4—C8	121.13 (10)	C13—C12—C11	109.86 (9)
C3—C4—C8	121.28 (10)	C13—C12—H12A	109.7
C4—C5—C6	121.54 (10)	C11—C12—H12A	109.7
C4—C5—H5	119.2	C13—C12—H12B	109.7
C6—C5—H5	119.2	C11—C12—H12B	109.7
C5—C6—C1	120.19 (10)	H12A—C12—H12B	108.2
C5—C6—C7	120.94 (9)	C12—C13—C14	109.84 (9)
C1—C6—C7	118.74 (9)	C12—C13—H13A	109.7
O1—C7—N2	122.54 (10)	C14—C13—H13A	109.7
O1—C7—C6	121.44 (9)	C12—C13—H13B	109.7
N2—C7—C6	115.95 (9)	C14—C13—H13B	109.7
C4—C8—H8A	109.5	H13A—C13—H13B	108.2
C4—C8—H8B	109.5	C13—C14—C9	112.16 (9)
H8A—C8—H8B	109.5	C13—C14—H14A	109.2
C4—C8—H8C	109.5	C9—C14—H14A	109.2
H8A—C8—H8C	109.5	C13—C14—H14B	109.2
H8B—C8—H8C	109.5	C9—C14—H14B	109.2
N1—C9—N2	106.31 (8)	H14A—C14—H14B	107.9
N1—C9—C10	107.80 (8)		
			
C9—N1—C1—C2	152.24 (10)	C5—C6—C7—N2	−168.95 (9)
C9—N1—C1—C6	−30.19 (14)	C1—C6—C7—N2	15.21 (14)
N1—C1—C2—C3	179.98 (10)	C1—N1—C9—N2	51.63 (12)
C6—C1—C2—C3	2.41 (16)	C1—N1—C9—C10	169.95 (9)
C1—C2—C3—C4	0.84 (17)	C1—N1—C9—C14	−68.21 (12)
C2—C3—C4—C5	−2.60 (16)	C7—N2—C9—N1	−42.79 (13)
C2—C3—C4—C8	177.80 (11)	C7—N2—C9—C10	−159.42 (9)
C3—C4—C5—C6	1.10 (16)	C7—N2—C9—C14	78.03 (12)
C8—C4—C5—C6	−179.30 (10)	N1—C9—C10—C11	172.15 (9)
C4—C5—C6—C1	2.13 (16)	N2—C9—C10—C11	−72.16 (11)
C4—C5—C6—C7	−173.64 (10)	C14—C9—C10—C11	49.90 (12)
N1—C1—C6—C5	178.45 (9)	C9—C10—C11—C12	−53.09 (12)
C2—C1—C6—C5	−3.87 (16)	C10—C11—C12—C13	57.52 (12)
N1—C1—C6—C7	−5.69 (15)	C11—C12—C13—C14	−60.01 (12)
C2—C1—C6—C7	171.99 (9)	C12—C13—C14—C9	58.30 (12)
C9—N2—C7—O1	−171.57 (10)	N1—C9—C14—C13	−172.67 (8)
C9—N2—C7—C6	11.38 (14)	N2—C9—C14—C13	69.67 (11)
C5—C6—C7—O1	13.96 (15)	C10—C9—C14—C13	−52.60 (11)
C1—C6—C7—O1	−161.87 (10)		

### Hydrogen-bond geometry (Å, °)

**Table:**

*D*—H···*A*	*D*—H	H···*A*	*D*···*A*	*D*—H···*A*
N2—H2A···O1^i^	0.901 (16)	2.058 (16)	2.9563 (13)	174.5 (13)

Symmetry codes: (i) −*x*, −*y*+1, −*z*+1.
